# Successful Management of C3 Glomerulopathy Recurrence Post-Kidney Transplantation with Iptacopan: A Case Report

**DOI:** 10.3390/ijms26115053

**Published:** 2025-05-24

**Authors:** Dario Troise, Barbara Infante, Silvia Mercuri, Michele Rossini, Loreto Gesualdo, Giovanni Stallone

**Affiliations:** 1 Nephrology, Dialysis and Transplantation Unit, Advanced Research Center on Kidney Aging (A.R.cK.A), Department of Medical and Surgical Sciences, University of Foggia, 71122 Foggia, Italy; barbara.infante@unifg.it (B.I.); silvia.mercuri1990@gmail.com (S.M.); giovanni.stallone@unifg.it (G.S.); 2 Renal Medicine and Baxter Novum, Department of Clinical Science, Intervention and Technology, Karolinska Institutet, 141 52 Stockholm, Sweden; 3 Unit of Nephrology, Dialysis and Transplantation, Department of Precision and Regenerative Medicine and Ionian Area (DiMePRe-J), University of Bari “Aldo Moro”, 70124 Bari, Italy; michelerossini@libero.it (M.R.); loreto.gesualdo@uniba.it (L.G.)

**Keywords:** C3 glomerulopathy, Iptacopan, complement system, innate immunity

## Abstract

C3 glomerulopathy (C3G) is the predominant cause of complement-mediated membranoproliferative glomerulonephritis and is considered a rare disorder caused by genetic or acquired dysregulation of the alternative complement pathway. There are no established treatment guidelines for treating kidney-transplanted recipients with C3G recurrence, as they are already on immunosuppressive protocols. Furthermore, non-complement-specific immunosuppressive drugs appear to offer limited benefits for patients with C3G in native kidneys. Therefore, modulating the complement system appears to be the most effective strategy for this specific patient population. We describe the use of Iptacopan in a 38-year-old kidney-transplanted patient with C3G recurrence. Iptacopan was associated with a significant and striking improvement in the patient’s clinical and laboratories status. A follow-up kidney biopsy performed 5 months after the initiation of Iptacopan revealed a reduction in endocapillary, extracapillary and mesangial hypercellularity, along with a decreased extent of parietal proteinaceous deposits observed on light microscopy. The direct control of the complement dysregulation underlying the pathogenesis of C3G with Iptacopan was accompanied by improvements in clinical, laboratory and histological features, with demonstrated reduced disease activity and slowed disease progression. Therefore, the case report described is intended to shed light on the potential role of new AP complement blockers in the treatment of C3G.

## 1. Introduction

Membranoproliferative glomerulonephritis (MPGN) refers to a histological pattern of glomerular injury characterized by mesangial hypercellularity and thickening of the glomerular basement membrane with double contours and cellular interposition, identifiable by light microscopy and associated with immune complexes and/or complement factors deposition. Despite the histopathologic pattern is distinctive, diagnosis is complicated by immunofluorescence and electron microscopy, which can identify different forms of MPGN [1]. Originally, MPGN was classified into types I, II and III based on electron microscopy findings. Nowadays, the classification is based on immunofluorescence findings and is linked to the underlying disease pathogenesis, allowing to identify immune complex-mediated MPGN, complement-mediated MPGN and MPGN with negative pattern for both immune complexes and complement components. C3 glomerulopathy (C3G) accounts for the majority of complement-mediated MPGN [2], and it is considered a rare disorder caused by genetic or acquired dysregulation in one or more of the multiple components of the alternative complement pathway (AP), histopathologically marked by the accumulation of the C3 complement factor in kidney tissue in the near absence of immunoglobulin deposits and with classic clinical features of glomerulonephritis [3]. The incidence is approximately 1–2 cases per million people each year, and nearly 50% of patients progress to kidney failure within 10–15 years from the diagnosis. The clinical course includes asymptomatic urinary abnormalities, nephrotic or nephritic syndromes, severe acute kidney injury and progressive chronic kidney disease. Unfortunately, while kidney transplantation is considered a viable replacement therapy, the disease is associated with significantly high recurrence rates and allograft failure [4].

There is no established mainstay of therapy or guidelines for treating kidney-transplanted patients with C3G recurrence, since these patients are already on immunosuppressive protocols, including corticosteroids, mycophenolate mofetil (MMF) and calcineurin inhibitors (CNIs). Moreover, these non-complement-specific immunosuppressive drugs appear to offer limited benefits for patients with C3G in native kidney [5,6,7]. Therefore, modulating the complement system appears to be the most effective strategy for this specific patient population. 

We describe the clinical use of Iptacopan in a kidney-transplanted patient with C3G recurrence. Iptacopan is a complement factor B-targeting drug currently in phase II and III of development for several kidney-involved diseases, including C3G [8].

## 2. Case Report

A 38-year-old White male kidney transplant recipient, with a history of histologically proven C3 glomerulopathy in his native kidneys, presented with nephrotic syndrome. Physical examination revealed blood pressure of 160/95 mmHg, a heart rate of 80 bpm, periorbital edema and severe peripheral pitting edema. The rest of the examination was unremarkable. When asked about other symptoms, the patient reported asthenia, reduced urinary output and an increase in body weight (8 kg over the past 3 weeks). Laboratory studies revealed worsening renal function (serum creatinine level, 2.6 mg per deciliter vs. 1.38 mg per deciliter at the time of discharge from the Transplant Center at the age of 27 years), elevated proteinuria (22.7 g per day), and marked hypoalbuminemia (2.6 g per deciliter). Urinalysis revealed microscopic hematuria and glycosuria. The patient’s white blood cells and platelets count were within normal limits, as well as coagulation parameters, bleeding time, serum electrolytes (except for a mild hyperkalemia), C-reactive protein and liver and pancreatic functions. No abnormal ultrasonographic findings were noted, with homogeneous blood flow observed throughout the transplant kidney on color Doppler ultrasound (mean Resistive Index 0.70). Moreover, no immunological evidence of allograft rejection was identified, as indicated by the absence of anti-HLA antibodies.

Interestingly, the patient’s medical history includes two episodes of nephrotic syndrome, immediately and approximately six years after transplantation, not histologically confirmed, managed at a different facility with increased immunosuppression therapy, which led to a reduction in proteinuria to less than 1 g per day.

Additionally, further investigation into the causes of nephrotic syndrome revealed negative autoimmune serologies, including ANA, ANCA, anti-dsDNA, anti-GBM and reduced serum C3 levels (66 mg per deciliter; normal range: 90–180 mg per deciliter), while C4 levels remained within normal range. Moreover, no clinical, laboratory or serological evidence of any infections was identified. The serum IgG, IgA and IgM concentrations were markedly reduced (42, 38 and 38 mg per deciliter, respectively). Electrophoresis did not show the presence of monoclonal components.

**Figure 1:**
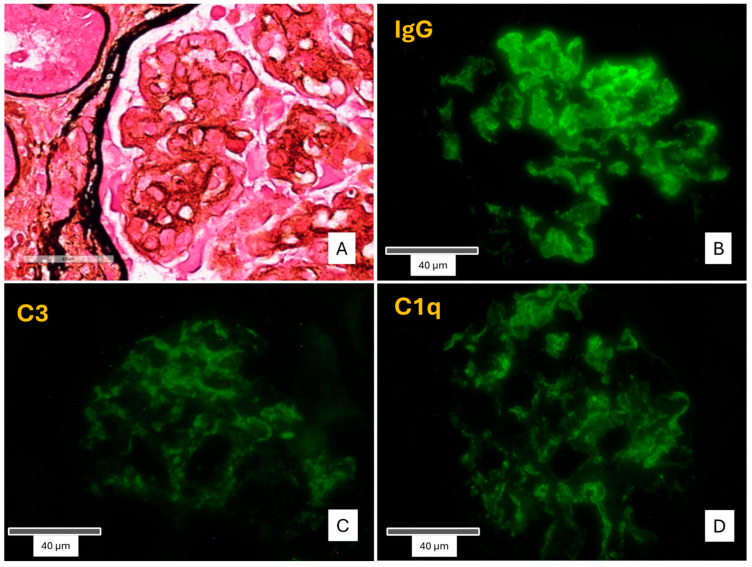
Pathological findings of the first kidney biopsy. (**A**): Membranoproliferative pattern. Double contours of the glomerular basement membranes with cellular interposition and proteinaceous subendothelial deposits (arrows). (Methenamine Silver). (**B**): IgG deposits in the mesangium and, globally distributed, along the glomerular basement membranes, coarsely granular, “sausage”-shaped (anti-IgG,×400). (**C**,**D**): C3 and C1q deposits in the mesangium and along the glomerular basement membranes.

After explaining the benefits and potential risks and obtaining the patient’s written informed consent, a renal biopsy of the transplanted kidney was performed. Light microscopy demonstrated the presence of 19 glomeruli in total, 2 of which were globally sclerotic. The remaining glomeruli showed a lobular appearance of the glomerular tuft, along with four crescents (two cellular, one fibrocellular and one fibrotic) and proteinaceous subendothelial deposits. The basement membranes showed segmental duplication and a characteristic “tram track” appearance, associated with cellular interposition and diffuse endocapillary hypercellularity. Subendothelial and mesangial deposits were also observed. Immunofluorescence testing was positive for diffuse finely granular deposits of C3, C1q, and coarsely granular, “sausage”-shaped polyclonal IgG, along the glomerular capillary walls and the mesangium. The conclusive diagnosis was “recurrence of Type I MPGN” [Figure 1]. 

The activity score of the C3G histologic index was 13 [range: 0–21].

Further analysis of the complement components and genetic mutations were performed revealing the presence of both properdin-independent C3 nephritic factor (C3NeF) and C5 nephritic factor (C5NeF).

Intravenous high-dose methylprednisolone was administered as bolus dose of 1 gr/die for three consecutive days, followed by a gradual tapering regimen, aiming to modulate glomerular inflammation and suppress the production of complement-activating autoantibodies.

Progressive decline in urine output and significant weight gain led to the initiation of continuous renal replacement therapy (CRRT) on day six, using continuous veno-venous hemofiltration (CVVH). However, due to the lack of clinical improvement, five consecutive daily plasma exchanges (PEX) were initiated on day eleven.

He had ongoing episodes of dyspnea and hemoptysis, accompanied by fever and abdominal pain on day seventeen, which required oxygen therapy and radiological evaluation. Imaging revealed abdominal and thoracic effusion, along with lung consolidation. Antimicrobial therapy was initiated, and RRT continued. Laboratory testing for infections revealed an active cytomegalovirus (CMV) infection on day twenty-three. Therefore, the patient was treated with Valganciclovir and immunosuppressive therapy was reduced by discontinuing mycophenolic acid (MMF), leading to subsequent resolution of the infection.

**Figure 2:**
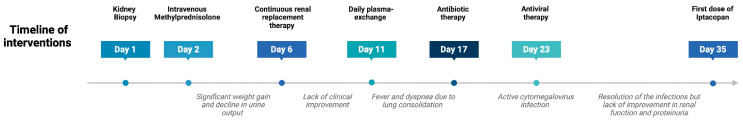
Timeline of interventions before first dose of Iptacopan. The figure illustrates timeline and clinical course between interventions and Iptacopan initiation. Created in BioRender. Troise, D. (2025), https://BioRender.com/z1hiq62 (accessed on 19 May 2025).

Given the histological findings, the presence of autoantibodies activating the complement cascade, and the lack of response to the previous treatments, compassionate use of the drug Iptacopan (Novartis Pharmaceuticals) was requested. On the morning of day thirty-five, after the recommended vaccinations, he received his first dose of Iptacopan [Figure 2].

## 3. Results

The initiation of Iptacopan was associated with a significant and striking improvement in the patient’s clinical and laboratories status, including no more requirement of hemodialysis treatment. The patient’s blood pressure and clinical signs of fluid overload were well controlled with antihypertensive and diuretic therapy. 

Serum creatinine levels initially increased, reaching levels of 3.5 mg per deciliter after 1 month and then decreased to 2.17 mg per deciliter within 3 months of treatment. 

A substantial reduction in proteinuria was observed. Initial levels of 22.7 gr per day progressively decreased to 4.68 gr per day at 1 month and to 0.69 gr per day at 3 months of treatment. Notably, proteinuria measurements were performed on urine samples with comparable diuresis volumes, ensuring reliable assessment. 

Serum C3 levels, which were markedly depressed at baseline, showed substantial improvement over time, reaching physiological range levels within 1 month of treatment, and improving to 154 mg per deciliter within 3 months. 

**Figure 3:**
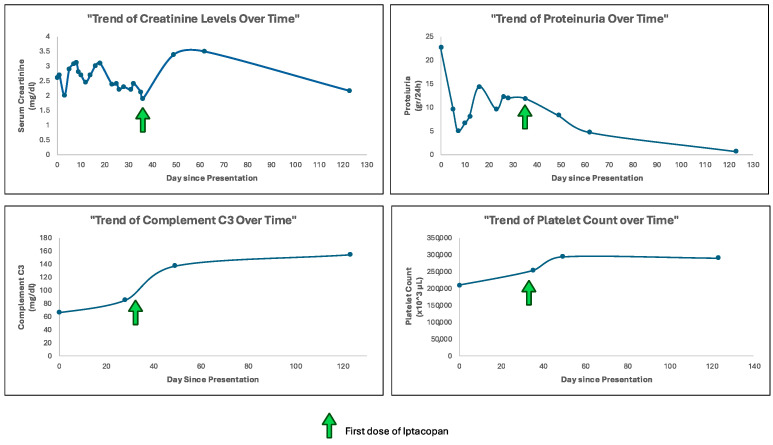
Laboratory measures before and after the first dose of Iptacopan. The figure illustrates the trajectories of key laboratory values for the subject of the study in relation to the first administration of Iptacopan. The vertical green arrow indicates the initiation of Iptacopan treatment at a dose of 200 mg administered orally twice a day.

Thrombocytopenia is considered one of the common adverse events associated with the use of Iptacopan. However, in our case, platelet count did not decrease throughout the treatment period (mean value 261,500 ± 34.26 per microliter) [Figure 3].

All of the other laboratory parameters remained stable. No significant adverse events related to Iptacopan were reported.

**Figure 4:**
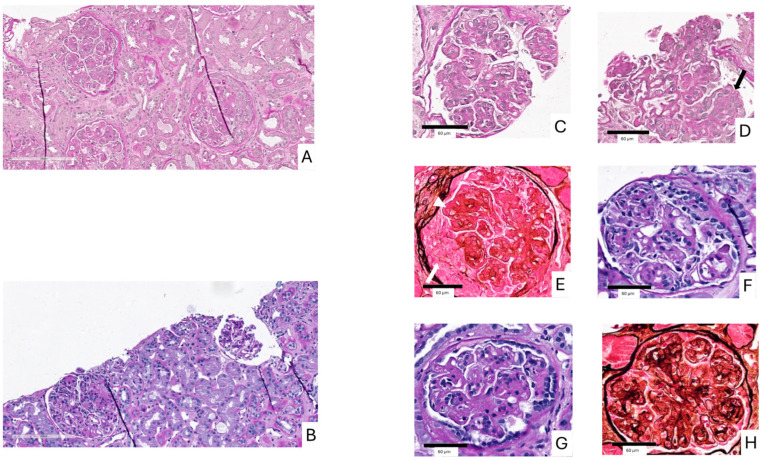
(**A**,**B**) Comparison between the first kidney biopsy and the follow-up biopsy at low magnification. (**A**): Low magnification from the first biopsy showing diffuse MPGN pattern of glomerular injury along with diffuse endocapillary proliferation and extracellular proliferation with cellular crescent formation (Periodic Acid Shiff). (**B**): Low magnification from the second biopsy showing persistence of MPGN pattern of glomerular injury with slightly less endocapillary and mesangial hypercellularity. (**C**–**H**) Comparison between the first kidney biopsy and the follow-up biopsy at high magnification. (**C**–**E**): High magnification from the first biopsy showing diffuse MPGN pattern of glomerular injury along with diffuse endocapillary proliferation (black arrow) and extracellular proliferation with cellular crescent formation (white arrow). Proteinaceous material is observed in the subendothelial region (arrowheads). (**C**,**D**): Periodic Acid Shiff, (**E**) Methenamine Silver). (**F**–**H**): High magnification from the second biopsy showing persistence of MPGN pattern of glomerular injury with slightly less endocapillary and mesangial hypercellularity.

A follow-up kidney biopsy performed 5 months after the initiation of Iptacopan revealed a reduction in endocapillary, extracapillary and mesangial hypercellularity, along with a decreased extent of parietal proteinaceous deposits observed on light microscopy. Additionally, decreased C3G histology activity score to 9 was observed [Figure 4].

## 4. Discussion

The knowledge of the clinical presentation of C3G recurrence after kidney transplantation is primarily based on a small number of retrospective studies, resulting in limited insight into the pathological features of the disease in this specific patient population [9,10,11]. We described the use of Iptacopan, as a novel salvage therapy in a patient with native kidney failure attributed to C3G, who experienced disease recurrence after kidney transplantation and showed clinical deterioration despite daily plasma exchange and the intensified immunosuppressive regimen. The administration of Iptacopan was followed by improvement of the patient’s clinical condition and resulted in a reduction in proteinuria and serum creatinine levels.

When a membranoproliferative pattern of injury is observed on a kidney biopsy, it does not identify a specific disease; therefore, further characterization of the disease beyond its histological features is recommended, as suggested by the KDIGO 2021 guideline for the management of glomerular diseases. Concordantly, an appropriate evaluation of the complement system should be undertaken if immunofluorescence microscopy reveals a complement-dominant pattern, suggesting the possibility of C3 or C4 glomerulopathy. Furthermore, before confirming a diagnosis of C3G, concurrent or prior infections and the presence of a monoclonal component should be excluded, as they require distinct therapeutic approaches [12]. According to these recommendations, we excluded the presence of infections, monoclonal gammopathy and autoimmune serological markers. Moreover, a thorough evaluation to identify genetic abnormalities in the complement system was performed, showing the presence of both C3NeF and C5NeF, confirming a dysregulation of the complement cascade.

Recently, a case series of C3G showed that the disease is frequently characterized by recurrence in the kidney allograft despite the use of immunosuppression [11]. Additionally, it was shown that recurrence typically occurs very early after kidney transplantation, within the first 4 months, usually with minimal proteinuria and mild histologic alterations [13]. Interestingly, and in contrast to what is observed in the scientific literature, our patient developed a C3G recurrence approximately 11 years after kidney transplantation, a significantly delayed onset compared to the early recurrence commonly described. Delayed recurrence can occur due to subtle complement dysregulation that remains clinically silent for years. Potential triggers for late recurrence may include infections, immunologic shifts, or changes in immunosuppressive therapy. However, in this particular case, no specific event was identified highlighting the importance of long-term monitoring even many years after transplantation, as late recurrence remains a possible clinical event. Notably, the two episodes of nephrotic syndrome that occurred shortly after transplantation could have been attributable to disease recurrence. However, in the absence of histological evidence, this remains speculative. Therefore, post-transplant monitoring and implementation of the available therapies are advisable to improve clinical outcomes [10].

Complement dysregulation is the primary driver of C3G, and targeted complement inhibitors for this disease have shown varying levels of success. Although anti-complement targeting therapies are currently under investigation and no complement inhibitor has been approved yet for the management of C3G, Eculizumab, a terminal complement blocker, has been used on an off-label basis as an alternative option for patients with progressive disease who do not respond to other treatment. However, the benefit of Eculizumab in the management of patients with native and post-transplant C3G appears to be limited, with only some patients achieving a sustained response to treatment [11,14,15]. Moreover, in the ACCOLADE trial, the effect of Avacopan, an oral C5a receptor inhibitor, was evaluated in patients with native and post-transplant disease, demonstrating only limited efficacy in reducing C3G progression [16]. This limitation could be explained by the fact that the block of the complement system happens on the downstream of the complement cascade and the cornerstone of C3G pathogenesis seems to be an increase in AP C3 convertase formation, making this upstream axis the main target for the development of new therapies [3]. 

Recently, Wong et al. evaluated proximal complement inhibition with Iptacopan in a phase 2, multicenter study for patients with biopsy-proven native kidney C3G and kidney transplant recipients with C3G recurrence. The authors demonstrated a significant decrease in C3 staining in the recurrent transplant cohort after approximately 3 months of treatment compared to baseline, meeting the primary endpoint of the study. Moreover, serum C3 levels were normalized in most patients. However, no significant reduction in proteinuria was observed, unlike our patient who achieved the resolution of proteinuria [17]. The improvement in proteinuria observed in our patient is a notable finding, particularly given the limited data on post-transplant recurrence, although findings from an individual case may not be directly applicable to larger trial populations. The heterogeneity of C3 glomerulopathy, driven by distinct genetic or acquired factors affecting complement system regulation, as well as variations in the immunologic milieu and time from kidney transplantation may contribute to differential responses to factor B inhibition with iptacopan.

**Figure 5:**
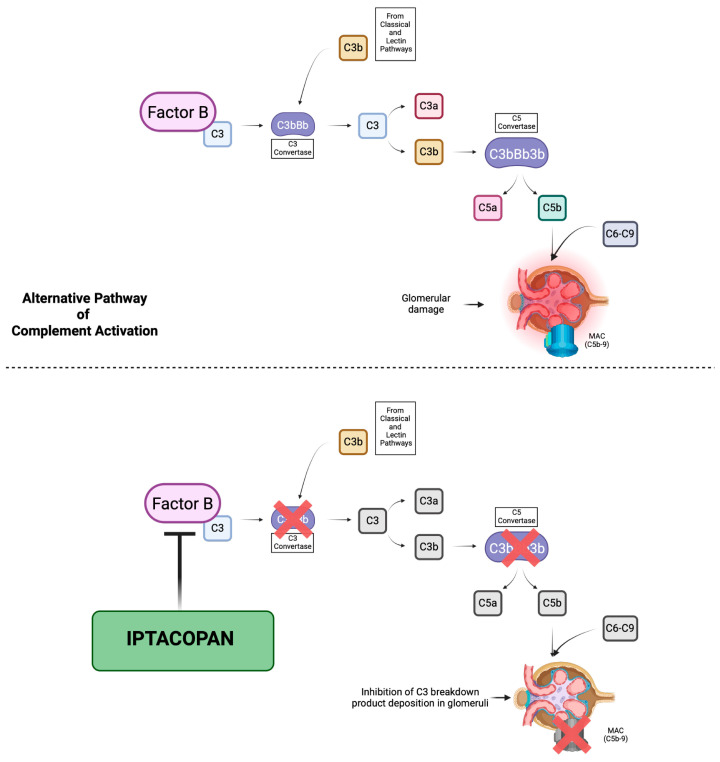
Iptacopan mechanism of action. The initiation of the alternative pathway (AP) occurs spontaneously via a process known as “tickover”, where the protein C3 undergoes a low rate of hydrolysis in the fluid phase. Subsequently, C3 binds to factor B, a key component of the alternative pathway, which is then cleaved into Ba and Bb, leading to the formation of the AP C3 convertase (C3Bb). Additionally, C3b from Classical and Lectin pathways can contribute to the amplification of the AP, facilitating cross-talk between pathways. Then, C3b leads to the generation of C5 convertases, which cleave C5 into C5a and C5b. C5b initiates the assembly of the membrane attack complex (MAC), a structure composed of C5b, C6, C7, C8 and C9. The MAC forms transmembrane pores on the cell surfaces, ultimately leading to cell lysis. The mechanism of action of Iptacopan is based on the inhibition of the enzymatic activity of factor B. This inhibition suppresses the formation of both C3 and C5 convertases, thereby regulating the AP at both upstream (C3 activation) and downstream (C5 activation) levels. As a result, the generation and subsequent deposition of C3 breakdown products in the glomeruli and the formation of the MAC are effectively prevented. Created in BioRender. Troise, D. (2025), https://BioRender.com/arm1qbp (accessed on 14 May 2025).

The mechanism of action of Iptacopan is based on the inhibition of the enzymatic activity of the serine protease factor B and therefore the cleavage of C3 and C5, which leads to the suppression of both convertases, resulting in both upstream and downstream regulation of the AP and preventing the further deposition of C3 breakdown products in the glomerular compartment, targeting the complement dysregulation that underlies C3G [18] [Figure 5]. In our patient, 5 months of treatment with Iptacopan led to a reduction in the endocapillary, extracapillary and mesangial hypercellularity on the renal biopsy. These changes were accompanied by an increased serum C3 levels and the resolution of proteinuria. Moreover, we applied the C3G histologic index, a tool proposed for the assessment of C3G outcomes by the evaluation of seven parameters of diseases activity using a semiquantitative scale of 0–3, allowing for a total activity score range from 0 to 21 [19,20]. In our case, we observed a reduction in the C3G histology activity score from 13 to 9. Additionally, Iptacopan was well tolerated, without significant treatment-emergent adverse events.

## 5. Methods

After obtaining informed consent, Iptacopan (Novartis Pharmaceuticals) was administered at a dose of 200 mg orally twice a day, based on the available clinical trial protocols designed to evaluate the efficacy and safety of Iptacopan in C3G. The treatment began on day thirty-five and continued without interruption. During this time, the patient was closely monitored for efficacy and safety.

As part of his ongoing immunosuppressive regimen for the transplanted kidney, the patients was on prednisone 5 mg/day, tacrolimus (T.L. 8 ng/mL) and MMF 180 mg twice a day, which was reintroduced at the resolution of the CMV infection.

Moreover, renoprotective strategies, including antiproteinuric therapy (Irbesartan 150 mg/die), antipertensive drugs (carvedilol 12.5 mg twice a day), diuretics (furosemide 25 mg twice a day and Hydrochlorothiazide 25 mg/die) were assessed. Metabolic disturbances were managed with dapagliflozin 10 mg/die and atorvastatin 20 mg/die. These therapies were implemented to slow renal disease progression and were continued or adjusted as needed based on the patient’s clinical response and laboratory results during the treatment period with Iptacopan.

The patient was closely monitored for renal function, including serum creatinine, urea and proteinuria (evaluated using 24 h urine protein measurement), serum electrolytes, complement C3 and C4 levels, immunosuppressive drug levels, complete blood count, infections and sepsis markers. Moreover, clinical signs of fluid retention were also regularly assessed.

Monitoring was performed on a daily basis during the initial phase of the treatment during the hospital stay and then transitioned to weekly and progressively to monthly after stabilization. Moreover, both kidney biopsies were evaluated by a renal pathologist who was blinded to all clinical and laboratory data. The C3G histologic activity index scoring was then validated by the same blinded pathologist to ensure consistency and internal validation of the assessment.

## 6. Conclusions

The direct control of the complement dysregulation underlying the pathogenesis of C3G with Iptacopan was accompanied by improvements in clinical, laboratory and histological features, with demonstrated reduced disease activity and slowed disease progression. Therefore, the case report described is intended to shed light on the potential role of new AP complement blockers in the treatment of C3G. Unfortunately, the treatment of C3G remains unclear due to a lack of sufficient data from randomized controlled trials and inconsistent results with immunosuppressive and complement-targeted therapies [12]. Thus, while awaiting robust data from clinical trials, it is crucial to obtain insights from individual case reports to understand the efficacy and safety profile of new complement-modulating drugs, such as Iptacopan, which provide valuable preliminary information and guide clinical decisions for the management of this complex and challenging disease.

## Data Availability

The original contributions presented in this study are included in the article. Further inquiries can be directed to the corresponding author.
